# Progress Toward Global Eradication of Dracunculiasis — January 2017–June 2018

**DOI:** 10.15585/mmwr.mm6745a3

**Published:** 2018-11-16

**Authors:** Donald R. Hopkins, Ernesto Ruiz-Tiben, Adam J. Weiss, Sharon L. Roy, James Zingeser, Sarah Anne J. Guagliardo

**Affiliations:** ^1^The Carter Center, Atlanta, Georgia; ^2^Division of Parasitic Diseases and Malaria, Center for Global Health, World Health Organization Collaborating Center for Dracunculiasis Eradication, CDC.

Dracunculiasis (Guinea worm disease), caused by the parasite *Dracunculus medinensis*, is acquired by drinking water containing copepods (water fleas) infected with its larvae. The worm typically emerges through the skin on a lower limb approximately 1 year after infection, causing pain and disability (*1*). The worldwide eradication campaign began at CDC in 1980. In 1986, the World Health Assembly called for dracunculiasis elimination, and the global Guinea Worm Eradication Program (GWEP), led by the Carter Center in partnership with the World Health Organization (WHO), United Nations Children’s Fund (UNICEF), CDC, and others, began assisting ministries of health in countries with dracunculiasis. There is no vaccine or medicine to treat the disease; the GWEP relies on case containment* to prevent water contamination and other interventions to prevent infection, including health education, water filtration, chemical treatment of water, and provision of safe drinking water (*1*,*2*). In 1986, an estimated 3.5 million cases^†^ occurred each year in 20^§^ African and Asian countries (*3*,*4*). This report, based on updated health ministry data (*3*), describes progress during January 2017–June 2018 and updates previous reports (*1*,*4*). In 2017, 30 cases were reported from Chad and Ethiopia, and 855 infected animals (mostly dogs) were reported from Chad, Ethiopia, and Mali, compared with 25 cases and 1,049 animal infections reported in 2016. During January–June 2018, the number of cases declined to three cases each in Chad and South Sudan and one in Angola, with 709 infected animals reported, compared with eight cases and 547 animal infections during the same period of 2017. With only five affected countries, the eradication goal is near, but is challenged by civil unrest, insecurity, and lingering epidemiologic and zoologic questions.

Comparisons of the numbers of reported *D. medinensis* cases between years and countries have been made (Table 1). There was a 20% increase in cases in 2017 (30), compared with 2016 (25); cases were reported from two countries in 2017 (Chad and Ethiopia) compared with three countries in 2016 (Chad, Ethiopia, and South Sudan). During the first 6 months of 2018, seven cases in humans were reported from three countries, compared with eight cases during the same period of 2017, all from Chad. Similar comparisons for animal infections have been made (Table 2). During 2017, 855 animal infections were reported, an 18% decline, compared with the 1,049 infections reported in 2016; in both years, animal infections occurred only in Chad, Ethiopia, and Mali. During January–June 2018, 709 animal infections (mostly in dogs) were reported, compared with 547 during January–June 2017 (Table 2). *D. medinensis* worms removed from animals are genetically indistinguishable from those removed from humans (*5*).

**TABLE 1:** Number of reported indigenous dracunculiasis cases in humans,* by country — worldwide, January 2016–June 2018

Country	No. of cases (% Contained)		% Change	No. of cases (% Contained)		% Change
	Jan–Dec 2016	Jan–Dec 2017	Jan–Dec 2016 to Jan–Dec 2017	Jan–Jun 2017	Jan–Jun 2018	Jan–Jun 2016 to Jan–Jun 2017
Chad	16 (56)	15 (60)	-6	8 (75)	3 (100)	-63
Ethiopia	3 (67)	15 (20)	400	0	0	0
Mali^†^	0	0	0	0	0	0
South Sudan	6 (50)	0	-100	0	3 (0)	∞
Angola^§^	0	0	0	0	1 (0)	∞
**Total**	**25 (56)**	**30 (43)**	**20**	**8 (75)**	**7 (43)**	**-13**

* No international importations were reported during the 18-month period January 2017–June 2018.

^†^ Civil unrest and insecurity since a coup d'état in April 2012 continued to constrain program operations in regions with endemic dracunculiasis (Gao, Kidal, Mopti, and Timbuktu) during 2017–2018.

^§^ Final classification of case origin pending further investigation.

**TABLE 2:** Number of reported indigenous dracunculiasis infections in animals,* by country — worldwide, January 2016–June 2018

Country	No. of Cases (% Contained)		% Change	No. of Cases (% Contained)		% Change
	Jan–Dec 2016	Jan–Dec 2017	Jan–Dec 2016 to Jan–Dec 2017	Jan–Jun 2017	Jan–Jun 2018	Jan–Jun 2016 to Jan–Jun 2017
Chad	1,022 (65)	830 (75)	-19	535 (75)	696 (78)	30
Ethiopia	16 (63)	15 (40)	-6	10 (30)	10 (40)	0
Mali^†^	11 (73)	10 (80)	-9	2 (50)	3 (67)	0
**Total**	**1,049 (65)**	**855 (65)**	-18	**547 (73)**	**709 (78)**	**30**

* No international importations were reported during the 18-month period January 2017–June 2018.

^†^ Civil unrest and insecurity since a coup d'état in April 2012 continued to constrain program operations in regions with endemic dracunculiasis (Gao, Kidal, Mopti, and Timbuktu) during 2017–2018.

In affected countries, the national GWEP receives monthly case reports from each village under active surveillance^¶^ and calculates the proportion of villages reporting monthly (Table 3). Countries enter the WHO precertification stage of eradication after 1 full year with no reported indigenous cases. Villages where endemic transmission of dracunculiasis is interrupted (i.e., zero cases reported for ≥12 consecutive months) are kept under active surveillance for 2 additional consecutive years. WHO certifies a country free of dracunculiasis after maintenance of adequate nationwide surveillance for ≥3 consecutive years with no indigenous cases.** WHO has certified 199 countries, areas, and territories as free from dracunculiasis (*3*), with only seven countries still lacking certification: four where dracunculiasis remains endemic (Chad, Ethiopia, Mali, and South Sudan), one in the precertification stage (Sudan), and two that were never known to have had endemic dracunculiasis (Angola and the Democratic Republic of the Congo). In April 2018, while preparing for certification, Angola discovered an unexplained case that is still under investigation.

**TABLE 3:** Reported dracunculiasis cases in humans and animals, surveillance, and status of local interventions in villages with endemic disease, by country — worldwide, 2017

Cases in humans/Surveillance/Intervention status	Country				Total
	Chad*	Ethiopia	Mali^†^	South Sudan	
**Reported cases in human**					
No. of indigenous cases, 2017	15	15	0	0	**30**
No. of imported cases,^§^ 2017	0	0	0	0	**0**
% Contained^¶^ in 2017	60	20	0	0	**40**
% Change in indigenous cases in humans in villages or localities under surveillance, same period 2016 and 2017	-6	400	0	0	**20**
**Reported cases in animals**					
No. of indigenous cases, 2017	830	15	10	0	**855**
No. of imported cases,** 2017	0	0	5	0	**5**
% Contained^¶^ in 2017	75	40	80	0	**75**
% Change in indigenous cases in animals in villages or localities under surveillance, same period 2016 and 2017	-19	-6	-9	0	**-18**
**Villages under active surveillance, 2017**					
No. of villages	1,860	167	455	4,046	**6,528**
% Reporting monthly	99	98	99	99	**99**
No. of villages reporting ≥1 cases in humans	13	6	0	0	**19**
No. of villages reporting only imported** cases in humans	0	6	0	0	**6**
No. of villages reporting indigenous cases in humans	13	0	0	0	**13**
No. of villages reporting ≥1 cases in animals	271	6	10	0	**287**
No. of villages reporting only imported** cases in animals	0	0	5	0	**5**
No. of villages reporting indigenous cases in animals	271	6	5	0	**282**
**Status of interventions in villages with endemic human dracunculiasis, 2017**					
No. of villages with endemic human dracunculiasis, 2016–2017	20	9	0	4	**33**
% Reporting monthly^††^	100	100	—^§§^	100	**100**
% Filters in all households^††^	100	100	—^§§^	100	**100**
% Using temephos^††^	20	100	—^§§^	100	**52**
% ≥1 source of safe water^††^	80	89	—^§§^	50	**79**
% Provided health education^††^	100	100	—^§§^	100	**100**
**Status of interventions in villages with endemic animal dracunculiasis, 2017**					
No. of villages with endemic animal dracunculiasis, 2016–2017	378	9	15	0	**402**
% Reporting monthly^††^	100	100	100	0	**100**
% Using temephos^††^	18	100	100	0	**23**
% Provided health education^††^	100	100	100	0	**100**

* Participants at the annual Chad Guinea Worm Eradication Program review meeting in November 2014 adopted “1+ case village” as a new description for villages in Chad affected by cases of Guinea worm disease in humans or dogs infected with Guinea worms and defined it as “a village with one or more indigenous and/or imported cases of Guinea worm infections in humans, dogs, and/or cats in the current calendar year and/or previous year.”

^†^ Civil unrest and insecurity since a coup d'état in April 2012 continued to constrain program operations in regions with endemic dracunculiasis (Gao, Kidal, Mopti, and Timbuktu) during 2017–2018.

^§^ Imported from another country.

^¶^ Transmission from a patient with dracunculiasis is contained only if all of the following conditions are met for each emerged worm: 1) the infected patient is identified ≤24 hours after worm emergence; 2) the patient has not entered any water source since the worm emerged; 3) a village volunteer or other healthcare provider has managed the patient properly, by cleaning and bandaging the lesion until the worm has been fully removed manually and by providing health education to discourage the patient from contaminating any water source (if two or more emerging worms are present, transmission is not contained until the last worm is removed); 4) the containment process, including verification of dracunculiasis, is validated by a Guinea Worm Eradication Program supervisor within 7 days of emergence of the worm; and 5) temephos is used to treat potentially contaminated surface water if any uncertainty about contamination of these sources of drinking water exists, or if a such a source of drinking water is known to have been contaminated.

** Imported from another in-country village with endemic disease.

^††^ The denominator is the number of villages or localities with endemic disease where the program applied interventions during 2016–2017.

^§§^ Data are not available.

During January 2017–June 2018, CDC evaluated 120 worm specimens that emerged from humans, including 114 (95%) in countries with endemic dracunculiasis (47 from Chad, 17 from Ethiopia, and 50 from South Sudan), and six (5%) in countries not known to have currently endemic dracunculiasis (one from Angola, one from Cameroon, and four from the Democratic Republic of the Congo). Among the 120 specimens, 76 (63%) were from 2017 (27 [36%] identified as *D. medinensis* from Chad and Ethiopia) and 44 (37%) were from January–June 2018 (22 [50%] identified as *D. medinensis* from Angola, Chad, Ethiopia, and South Sudan). During 2017, 35 specimens from animals were submitted, 27 (77%) of which were identified as *D. medinensis*. The 27 confirmed worms came from Ethiopia (four baboons and 12 dogs), Mali (one cat and nine dogs), and Chad (one dog). During January–June 2018, 18 specimens from animals were submitted, and 17 (94%) were identified as *D. medinensis*, from Ethiopia (nine dogs), Mali (five dogs), and Chad (one cat, two dogs).

## Country Reports

**Chad**. After a decade with no reported cases, Chad reported 10 indigenous cases in humans in 2010. Dracunculiasis was declared to be endemic again in 2012 (*1*,*6*). Chad reported 15 cases in humans (10 contained) in 2017 and 16 cases (nine contained) in 2016. During the first half of 2018, Chad reported three cases in humans (all contained), compared with eight cases (six contained) during the same period in 2017 (Table 1). One of 14 villages reporting a case in a human in 2017 and none of three reporting a case during January–June 2018 had reported a case in a human previously.

In 2012, Chad reported Guinea worm infections in domestic dogs for the first time (*6*), primarily from communities along the Chari River. The Carter Center is assisting the Ministry of Health in implementing active village-based surveillance for human and animal infections in almost 1,900 at-risk villages. Because of previous investigations, a working hypothesis is that transmission in humans and dogs might occur without drinking contaminated water, perhaps through ingestion of fish or other aquatic animals that serve as transport or paratenic hosts (intermediate hosts in which the *D. medinensis* larvae live but do not develop). New infections can occur when humans consume inadequately cooked transport or paratenic hosts and when dogs eat such hosts raw (*6*). During 2017, a total of 817 domestic dog and 13 domestic cat infections were reported, 19% fewer than the 1,011 dog and 18% more than the 11 cat infections reported in 2016 (Table 2). This was the first observed reduction in infected dogs since 2012. However, during January–June 2018, there were 685 infected dogs and 11 infected cats reported, compared with 534 dogs and one cat during January–June 2017, representing a 30% increase.

Since October 2013, Chad’s GWEP urged villagers to cook their fish well, bury fish entrails, and prevent dogs from eating fish entrails. Since June 2017, approximately 81% of households sampled monthly in at-risk communities were burying fish entrails. In February 2014, health educators began persuading villagers to tether (contain) infected dogs until the worms emerged to prevent water contamination. In February 2015, the program introduced a reward equivalent to US$20 for reporting and tethering an infected dog. Whereas 40%, 68%, and 68% of infected dogs were tethered in 2014, 2015, and 2016, respectively, 76% of infected dogs were tethered in 2017, and 77% in January–June 2018.

Before 2010, Chad began offering a cash reward equivalent to US$100 for reporting a human dracunculiasis case. In areas under active surveillance, 80% of 885 residents surveyed during January–June 2018 knew of this reward, and 67% of 852 persons knew of the reward for reporting and tethering an infected dog. In July 2017, Chad launched a nationwide communication campaign to increase awareness of the cash rewards and knowledge about how to prevent dracunculiasis.

As of June 2018, 77% of villages reporting cases in humans had at least one source of drinking water free of copepods. Given limited use of temephos (an organophosphate larvicide used to treat unsafe water) in large lagoons used for fishing and for drinking water, a novel technique for applying temephos to cordoned sections of lagoons has been used to protect 19, 29, 61, and 57 villages in 2014, 2015, 2016, and 2017, respectively. Beginning October 2017, temephos also was applied monthly in small ponds in the villages with the most infected dogs, reaching 18 villages by December 2017 and 67 villages by June 2018.

The Carter Center and CDC are supporting research to better understand the unusual current epidemiology of dracunculiasis in Chad, assess antihelminthic treatment of dogs to prevent maturation of worms, and study food sources and movements of dogs in an area of Chad with endemic disease. The International Task Force for Disease Eradication reviewed much of this work in October 2017 (*7*). In collaboration with researchers from the University of Georgia, this initiative has demonstrated that fish can serve as transport hosts for *Dracunculus* spp. in the laboratory (*8*) and that *D. medinensis* can use frogs as paratenic hosts (*9*,*10*). As further proof of the latter, a *Dracunculus* larva has been recovered from a wild frog in Chad (*10*). Anthelminthic treatments of dogs with avermectins^††^ have not been effective.

**Ethiopia**. During January–December 2017, Ethiopia reported 15 cases of dracunculiasis in humans (three contained), among residents from six villages in Gambella and Oromia Regions, all among migrant laborers from Oromia who drank water from a contaminated pond at a commercial farm in Abobo district of Gambella (Table 1). January 2017–June 2018 represented the first time in several years that no case in humans was reported from Gambella’s Gog district. Ethiopia reported 11 infected dogs (six contained) and four infected baboons in 2017, compared with 14 dogs and two baboons in 2016, all in Gog district. During January–June 2018, Ethiopia reported no cases in humans, eight infected dogs (four contained), no infected baboons, and two uncontained infected domestic cats, all in Gog district, compared with six infected dogs (three contained) and four infected baboons (none contained) during January–June 2017. Since 2017, The Carter Center has provided support to Ethiopian public health and wildlife authorities on a baboon-dog epidemiology and ecology project.

The Ethiopian Dracunculiasis Eradication Program has 167 villages under active surveillance and is applying temephos monthly to most water sources used by humans or animals in the at-risk area of Gog district. In 2018, Ethiopia increased its cash rewards for reporting a human dracunculiasis case to US$360 (up from US$100 in 2014) and for tethering an infected animal to US$40 (up from US$20 in 2016). In 2017, approximately 83% of persons surveyed in active surveillance areas (Gambella Region and formerly endemic Southern Nations, Nationalities and Peoples’ Region) but only 22% of persons in Oromia knew of the rewards. Ethiopia launched a nationwide communication campaign to increase knowledge of rewards and dracunculiasis prevention in 2017.

**Mali.** In 2017, Mali reported no human dracunculiasis cases for the second successive year and reported no cases during January–June 2018 (Table 1). Mali reported 11 infected dogs (eight contained) in 2016 and nine infected dogs (six contained) and one infected domestic cat (uncontained) in 2017. During January–June 2018, Mali reported three infected dogs (two contained), all detected in Segou Region, and no infected cats. Parts of Mali’s area of endemicity are inaccessible because of insecurity. Mali has 455 villages under active surveillance and offers a US$100 reward for reporting a case in a human and US$20 for reporting and tethering an infected animal. During 2017, Malian health staff members questioned 33,000 persons about dracunculiasis during immunization campaigns; 86% of persons living in areas of active surveillance were aware of the cash reward for reporting an infected person. During April–June 2018, 83% of 190 persons surveyed were aware of the reward for reporting cases in humans, and 71% knew of the reward for reporting infected animals. Mali has launched a nationwide communication campaign to increase awareness of the rewards and improve knowledge of preventive measures.

**South Sudan.** During 2017, South Sudan reported no cases of dracunculiasis in humans for the first time (Table 1); only one infected animal has been reported (a dog in the same household as an infected person reported in 2015). After 19 consecutive months with no reported cases, the program discovered three cases in May and June 2018. All three patients were cattle herders from migratory communities in a recently pacified area of Western Lakes State that has suffered communal violence and displacements in recent years. In 2017, the program had 4,046 villages under active surveillance and increased the reward for reporting a dracunculiasis case to approximately US$139 and again to approximately US$400 in 2018. A 2017 survey of 50,612 residents in two counties of Warrap and Western Bahr Al Ghazal found that 72% of persons knew of the reward. In October 2017, South Sudan launched a nationwide campaign to increase knowledge of dracunculiasis prevention and awareness of the reward and in April 2018 established a National Committee for Documentation of Dracunculiasis Elimination.

## Discussion

In 2017, two countries (Chad and Ethiopia) continued to report cases of dracunculiasis in humans and animals. One additional country (Mali), with no cases in humans detected for 2 years, reported infected dogs and cats. In 2018, cases in humans were again detected in South Sudan, where fighting and civil unrest had limited adequate access. South Sudan has had traditional waterborne transmission of dracunculiasis, only one animal infection, strong programmatic leadership, and strong political support by the government, all suggesting that endemic transmission can be halted if adequate security can be maintained.

The discovery in April 2018 of a single dracunculiasis case in southern Angola was unexpected, and its etiology is unclear. No previous dracunculiasis case had been reported or alleged to have been imported from Angola. The worm (confirmed as *D. medinensis* by CDC) occurred in a child who had never traveled outside her home area and was discovered during a nationwide search for dracunculiasis as Angola prepared for WHO certification that it had no endemic disease. Initial investigations found no other confirmed infection in a human or dog in the child’s home village and neighboring villages.

Continued transmission of Guinea worm infections to a small number of dogs and cats in Ethiopia and Mali and baboons in Ethiopia appears to be geographically limited in each country (i.e., to a section of Ethiopia’s Gog district and adjacent districts of Mali’s Mopti and Segou regions). Transmission in both countries now appears to be driven by infected dogs; infections in other species (e.g., humans, cats, and baboons) might be incidental. In 2017, human transmission remained interrupted in Mali for the second consecutive year and was absent in Ethiopia’s Gog district for the first time. In 2017, the aberrant outbreak among 15 humans in Ethiopia in Abobo district was traced to a single shared source of contaminated drinking water, underscoring the importance of the parasite’s reproductive potential in favorable settings. The ecologic study of dogs and baboons now underway in Ethiopia might help to explain the unusual epidemiologic pattern of residual Guinea worm infections in both countries. DNA studies show promise in tracing genetic lineages of the worms.

Transmission of Guinea worm infections to many dogs and few humans in Chad continues a peculiar pattern that remains consistent after more than 8 years, manifested as single cases in humans in new villages each year, with infections rarely occurring in the same village in successive years. Laboratory tests indicate no distinction between Guinea worms removed from humans and dogs. Research studies increasingly favor the hypothesis that the parasite’s life cycle in Chad involves a transport or paratenic host. As research continues, the program has intensified active surveillance, education of villagers, containment of infected dogs, burial of fish entrails, and application of temephos, giving priority to villages with the most infected dogs.

The final four countries with endemic *D. medinensis* infections are distinguished from the other 17 countries that had endemic disease in the past: South Sudan because of its long civil war, Mali because of insecurity limiting access in some areas with endemic disease, and Chad, Ethiopia, and Mali because of the infection’s unusual epidemiologic patterns there.

**Summary**

**What is already known about this topic?**

The number of cases of dracunculiasis (Guinea worm disease) has decreased from an estimated 3.5 million in 1986 to 30 in 2017. Emergence of Guinea worm infections in dogs has complicated eradication efforts.

**What is added by this report?**

The number of human dracunculiasis cases reported declined to seven cases in three countries (Angola, Chad, and South Sudan) during January–June 2018, while the number of infected animals reported stood at 709 during the same period.

**What are the implications for public health practice?**

Existence of infected dogs, especially in Chad, and impeded access because of civil unrest and insecurity in Mali and South Sudan are now the greatest challenges to interrupting transmission.

## Footnotes

* Transmission from a patient with dracunculiasis is contained only if all of the following conditions are met for each emerged worm: 1) the infected patient is identified ≤24 hours after worm emergence; 2) the patient has not entered any water source since the worm emerged; 3) a village volunteer or other health care provider has managed the patient properly, by cleaning and bandaging the lesion until the worm has been fully removed manually and by providing health education to discourage the patient from contaminating any water source (if two or more emerging worms are present, transmission is not contained until the last worm is removed); 4) the containment process, including verification of dracunculiasis, is validated by a Guinea Worm Eradication Program supervisor within 7 days of emergence of the worm; and 5) the approved chemical temephos (Abate) is used to treat potentially contaminated surface water if any uncertainty about contamination of the source of drinking water exists, or if such a source of drinking water is known to have been contaminated.

† A dracunculiasis case is defined as an infection occurring in a person exhibiting a skin lesion or lesions with emergence of one or more worms laboratory-confirmed at CDC as *D. medinensis*. Because *D. medinensis* has a 10–14 month incubation period, each infected person is counted as a case only once during a calendar year.

§ Originally 20 countries, but the former country of Sudan officially separated into two countries (South Sudan and Sudan) on July 9, 2011.

¶ Villages under active surveillance are those that have endemic dracunculiasis or are at high risk for importation. Active surveillance involves daily searches of households for persons or animals with signs of dracunculiasis. An imported case is an infection resulting from ingestion of contaminated water in a place other than the community where the case is detected and reported. Since 2012, no internationally imported cases have been reported.

** An indigenous case of dracunculiasis is defined as an infection consisting of a skin lesion or lesions with emergence of one or more Guinea worms in a person who had no history of travel outside their residential locality during the preceding year.

†† Avermectins are a group of related macrocyclic lactone derivative drugs with potent anthelminthic activity.
